# An Uncommon Site, a Common Foe: Isolated Hepatic Tuberculosis in a HIV Positive Adult

**DOI:** 10.7759/cureus.96553

**Published:** 2025-11-11

**Authors:** Kevin Joseph R, Nivedita Thass, Sonal Saxena

**Affiliations:** 1 Microbiology, Maulana Azad Medical College, New Delhi, IND

**Keywords:** extrapulmonary tuberculosis (eptb), hepatic tuberculosis, hiv/aids, liver abscess, m. tuberculosis complex

## Abstract

Extrapulmonary tuberculosis such as hepatic tuberculosis, while rare, is becoming increasingly prevalent, especially in HIV endemic regions such as India. Hepatobiliary tuberculosis manifests either as isolated hepatic or isolated biliary, or combined hepatobiliary tuberculosis. We present a 28-year-old male, a known case of HIV on anti-retroviral therapy, with a history of smoking, alcoholism, and intravenous drug use, presenting with abdominal pain and vomiting for the past one week, with associated fever and fatigue for the past two weeks. On examination, he had pallor, icterus, and a tender abdomen. Initial baseline investigations revealed anemia, thrombocytopenia, raised prothrombin time/international normalized ratio, and raised alkaline phosphatase. Ultrasound and contrast-enhanced computed tomography of the abdomen revealed multiple liver abscesses, which were drained surgically. Chest X-ray was normal. Ziehl-Neelsen (ZN) staining of the aspirate was positive for acid-fast bacilli, and the MGIT culture flagged positive in 15 days. MPT64 rapid assay of the MGIT broth was also positive, suggestive of Mycobacterium tuberculosis complex (MTBC). The CD_4_ count was 108/µl. The patient’s clinical condition improved on initiation of anti-tubercular therapy (ATT). Clinicians in TB and HIV endemic countries, such as India, must maintain a high index of suspicion, especially among young patients presenting with liver abscess, as prompt diagnosis and initiation of ATT will lead to a significant reduction in morbidity and mortality.

## Introduction

According to the WHO, tuberculosis (TB) is the second leading cause of infectious death and fifth overall with 29.43 deaths per 1 lakh population in India [1]. Extrapulmonary TB (EPTB) accounts for 16% of the total TB cases worldwide and 20-24% in India. Incidence rate of TB is high in places where HIV is more prevalent, as HIV suppresses the immune system, leading to re-activation of latent TB [2]. While the diagnosis and management of pulmonary TB (PTB) have seen considerable advances, the same cannot be said for EPTB [3]. The ambiguity of clinical features, need for invasive specimens for diagnosis, paucibacillary nature of the specimens, and varying responses to treatment pose a serious challenge in the diagnosis and treatment of EPTB, such as hepatic TB [3]. Hepatobiliary TB is rare and is usually secondary to pulmonary or gastrointestinal TB. Isolated hepatic TB accounts for around 1% of TB cases and less than 100 cases have been reported in the literature to date [4].

## Case presentation

A 28 year old male, known case of HIV on anti-retroviral therapy (ART) with history of smoking, alcoholism, and intravenous drug use, presented to the medicine emergency with complaints of pain in the right upper abdomen which was continuous, dull aching, of moderate intensity, non-radiating, and associated with multiple episodes of bilious, non-projectile vomiting for the past one week. He also complained of on-and-off fever and fatigue for the past two weeks. There was no history of TB or contact with any known case of TB, even after HIV diagnosis. On examination, he had pallor and icterus, his pulse rate was 112 bpm, respiratory rate was 20/min, blood pressure - 102/60 mmHg, SpO2 - 96%, and a GCS score of 15/15. His abdomen was distended and tender in the right hypochondrium, with no guarding or rigidity, and with normal bowel sounds.

Initial baseline investigations revealed anemia (hemoglobin - 9 g/dl), thrombocytopenia (platelet count - 42,000/µl), raised PT/INR (21 sec/1.76), raised alkaline phosphatase (272 IU/L), while his total leucocyte count (TLC), differential leucocyte count (DLC), and other liver and kidney function tests were normal. The patient was initially resuscitated with intravenous fluids and analgesics. Blood transfusion was done in view of low hemoglobin.

**Figure 1:**
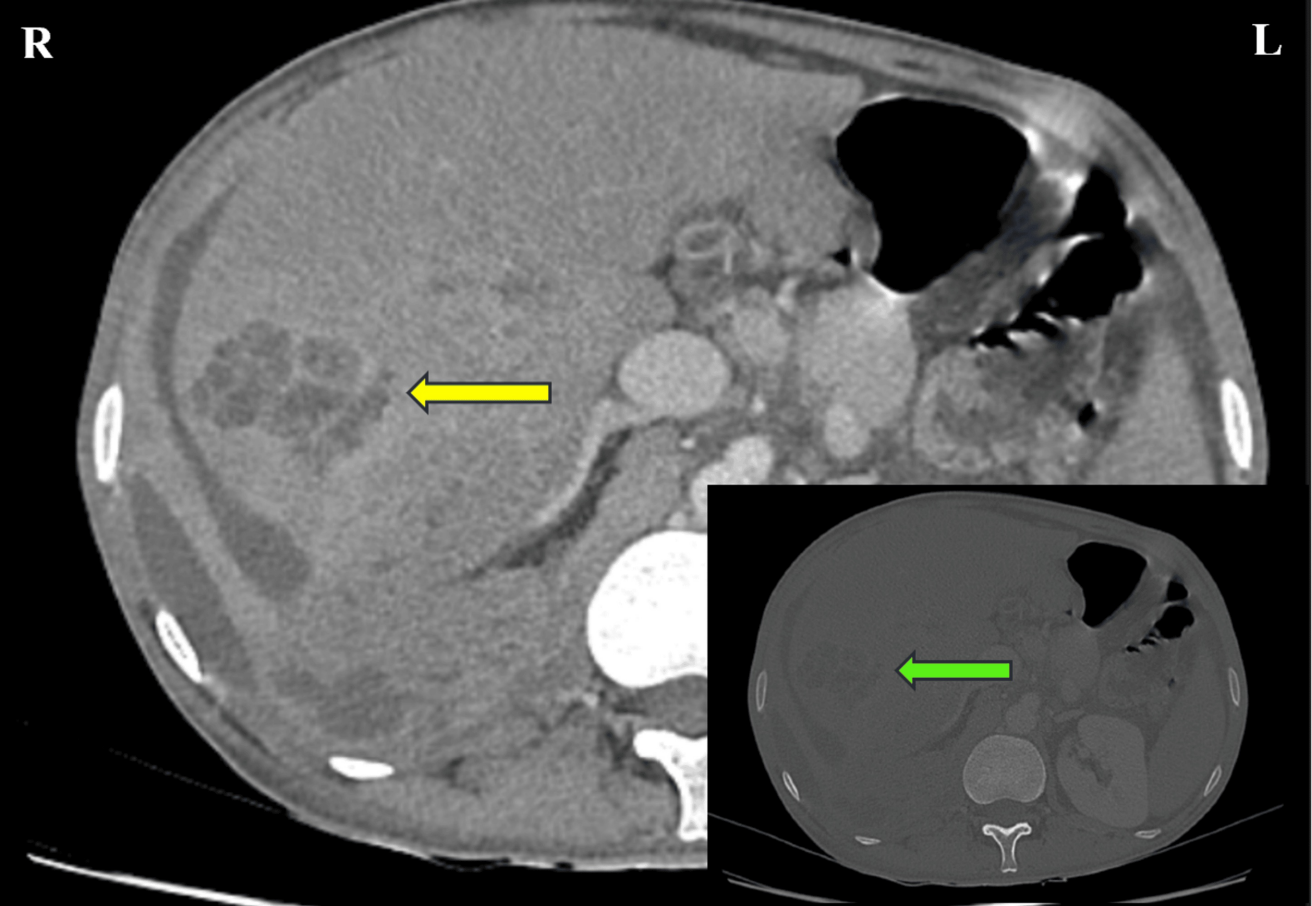
CECT Abdomen (coronal view) showing multiple, irregular hypodense areas within the liver parenchyma (yellow arrow); INSET – CECT abdomen thin bone (coronal view) showing multiple hypodense areas within the liver parenchyma (green arrow).

Ultrasound (USG) abdomen revealed an enlarged liver of size 16 cm, with two well-defined hypoechoic lesions, one in segment VII measuring 3.2x5.2 cm and another in segment V measuring 8.6x7.5 cm. There was also an associated perinephric collection, which was aspirated using an 18G needle. CECT abdomen showed multiple hypodense areas within the liver parenchyma (Figure 1). There were no findings suggestive of abdominal foci or lymphadenopathy. Chest X-ray was normal. The patient was provisionally diagnosed as a case of liver abscess and transferred to the surgery department for surgical drainage. The patient was empirically started on meropenem 3 grams intravenously thrice a day, vancomycin 2 grams intravenously twice a day, pantoprazole 80 milligrams intravenously twice a day, ondansetron 12 milligrams intravenously thrice a day, metronidazole 3 grams intravenously thrice a day, tablet pyridoxine 40 milligrams once a day, and tablet cotrimoxazole 960 milligrams once a day. USG-guided drainage of liver abscess with pigtail insertion was performed, and the sample was sent to the microbiology laboratory for investigation.

**Figure 2:**
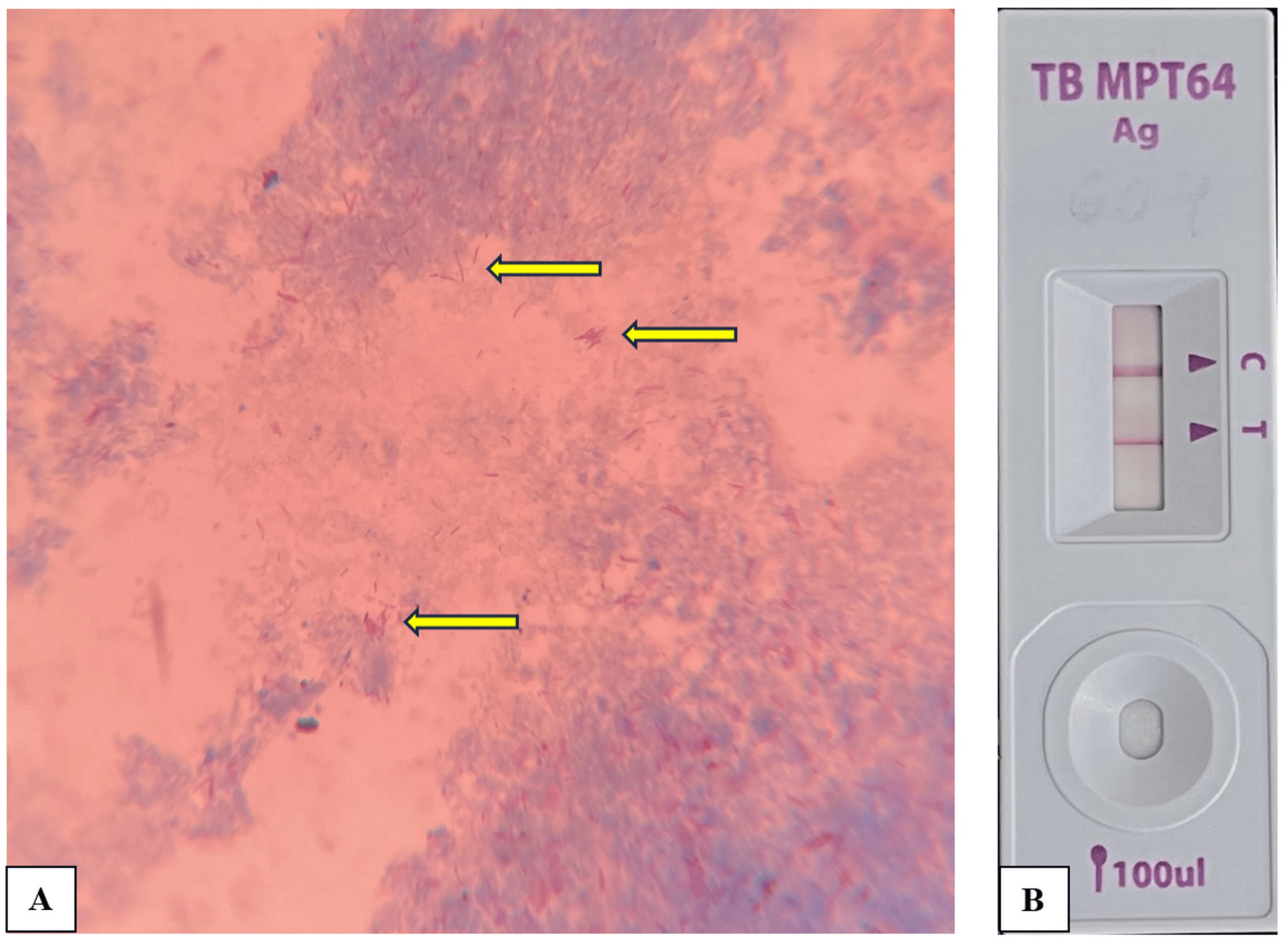
A – ZN staining of the liver aspirate showing multiple, pink colored, slender, beaded acid-fast bacilli (yellow arrow); B – MPT64 antigen rapid assay of the MGIT culture tube showing positive for M. tuberculosis complex (MTBC).

Bacterial culture yielded no growth after 48 hrs. On Ziehl-Neelsen (ZN) staining of the aspirate, multiple, pink-colored, slender, beaded acid-fast bacilli (AFB) suggestive of Mycobacterium tuberculosis (MTB) were observed (Figure 2A). Furthermore, culture using an automated mycobacterial liquid culture system (MGIT) flagged positive in 15 days. ZN staining from the positive MGIT broth also revealed multiple acid-fast bacilli, and the broth was also positive for MPT64 antigen by immunochromatography-based lateral flow assay (Figure 2B). Cartridge-based nucleic acid amplification test (CBNAAT) of the aspirate was negative for MTB. His CD4 count was 108/µl. His final diagnosis was tubercular liver abscess, and hence, he was subsequently initiated on anti-tubercular therapy (ATT) consisting of isoniazid, rifampicin, pyrazinamide, and ethambutol. Following clinical improvement, the pigtail was removed, and the patient was discharged subsequently.

Condition at the time of discharge: The patient was conscious, oriented, afebrile, pulse rate was 74 bpm, BP - 100/70 mmHg, SpO2 - 99% on room air. On discharge, the patient was advised to continue ATT and ART, prescribed tablet cotrimoxazole 960 milligrams once a day for five days, and requested to follow up in OPD at regular intervals.

## Discussion

Hepatobiliary tuberculosis, an uncommon presentation of Mycobacterium tuberculosis, manifests either as isolated hepatic or isolated biliary, or combined hepatobiliary tuberculosis. In regions such as India, tuberculosis is reported as the underlying cause in approximately 43% of hepatic granuloma cases [2]. A study in India among 18 hepatic TB cases observed that a preoperative diagnosis was made in only four (22.2%) cases, highlighting its diagnostic challenge [5]. HIV is a known risk factor for TB. Also, TB is the most common opportunistic infection and the leading cause of death among people living with HIV (PLHIV). In India, among PLHIV, 45-56% of all TB cases are extrapulmonary (EPTB) [6].

Tuberculosis of the liver and other forms of EPTB are usually seen in young adults with a mean age of 42 years (range: 19-72 years), while PTB has no such age predilection [5]. The most common clinical features in hepatic TB include pain in abdomen (40-83%), fever (30-100%), liver enlargement (10-100%), jaundice (0-60%), enlarged spleen (0-40%), and ascites (5-25%), of which abdominal pain, jaundice, and hepatomegaly were observed in our case as well [7]. A minuscule intestinal tubercular focus, which passes to the liver via the hepatic portal vein, is hypothesized to be the cause of isolated hepatic TB [8]. There was no abdominal foci or lymphadenopathy in our case.

Our case had raised alkaline phosphatase (ALP), while his aspartate aminotransferase (AST) and alanine aminotransferase (ALT) were normal. Previously, it has been noted that elevated ALP and GGT are more common in hepatic TB than elevated AST and ALT [7]. In addition, ALP levels are higher in hepatic tuberculosis among immunocompromised individuals compared to immunocompetent individuals [9]. Radiological features are generally non-specific and range from small hypoechoic nodules to large hypoechoic mass to hepatomegaly to abscesses, and hence are of little diagnostic value. Caseating granulomas are seen in up to 68% of tubercular liver abscess (TLA) cases, but are non-specific as such granulomas can be seen in other conditions like sarcoidosis [4].

Microbiological diagnosis could be made with various techniques such as microscopy, culture, and nucleic acid amplification test (NAAT). ZN stain is positive for AFB in around 45% of these cases, while it increases to 75% in PLHIV. The most specific test for the diagnosis of hepatic TB is culture of liver biopsy [4]. Our case was positive on microscopy and also grew in culture. CBNAAT, however, was negative in this case, possibly due to the paucibacillary nature of extrapulmonary specimens. In a previous study from India, the sensitivity and specificity of CBNAAT in diagnosing EPTB were 32.3% and 100%, respectively [10]. Some authors have reported a sensitivity and specificity of 53% and 96% respectively, when NAAT is done using liver biopsy specimen [4].

Depletion of CD4 helper T cells is a trademark of HIV infection, leading to reactivation of latent TB as cell-mediated immunity (CMI) declines. In fact, the rate of reactivation increases as the CD4 count falls, especially when the count falls below 200/µl [6]. The CD4 count in our case was 108/µl. Similarly, a case series on hepatic TB among HIV adults observed a median CD4 count of 47 cells/μl (inter-quartile range 27-107 cells/μl) [11]. The low CD4 count in our case was due to the poor adherence of the patient to the ART regimen. Suppression of viral replication is seen only when the compliance rate is > 95%. Earlier studies have observed that better compliance to ART is associated with CD4 counts of > 500 cells/µl [12]. Thus, the importance of strict adherence to ART cannot be understated.

Antitubercular therapy is the linchpin for treatment, with a standard regimen as prescribed by the national tuberculosis elimination program (NTEP) guidelines. Minimum duration of treatment is six months, but can be extended as per the treating physician’s discretion. As drugs such as isoniazid, rifampicin and pyrazinamide are hepatotoxic, treatment regimens may need to be curated on an individual level, based on the severity of liver dysfunction using scoring systems such as the Child-Turcotte-Pugh score and MELD score [4].

## Conclusions

EPTB, such as hepatic tuberculosis, is a rare entity that can manifest either as isolated hepatic or isolated biliary or combined hepatobiliary tuberculosis. The predicament in the diagnosis and treatment of TLA is due to the ambiguity of clinical features, the need for invasive specimens for diagnosis, the paucibacillary nature of the specimens, and varying responses to treatment. While hepatic tuberculosis responds well to standard ATT regimens, modifications may need to be done based on the severity of liver dysfunction.

Clinicians in TB and HIV endemic countries such as India must maintain a high index of suspicion especially in young patients presenting with liver abscess, as prompt diagnosis and ATT initiation lead to a significant reduction in morbidity and mortality. In addition, education and counselling on the importance of strict ART compliance in order to revert CD4 counts to normal levels is paramount.
